# E. coli Microcosms Indicate a Tight Link between Predictability of Ecosystem Dynamics and Diversity

**DOI:** 10.1371/journal.pgen.0020103

**Published:** 2006-07-14

**Authors:** Marianne Imhof, Christian Schlötterer

**Affiliations:** Institut für Tierzucht und Genetik, Wien, Austria; National Institute of Genetics, Japan

## Abstract

The diversity-stability hypothesis proposes that ecosystem diversity is positively correlated with stability. The impact of ecosystem diversity is, however, still debated. In a microcosm experiment using diverged Escherichia coli cells, we show that the fitness of community members depends on the complexity (number of participants) of the system. Interestingly, the spread of a community member with a superior genotype is mostly stochastic in low-complexity systems, but highly deterministic in a more complex environment. We conclude that system complexity provides a buffer against stochastic effects.

## Synopsis

The impact of diversity loss on the stability of ecosystems is a central issue in ecology. In today's world the continuous reduction in number of species, subspecies, and locally adapted populations, often with anthropogenic causes, turns it into a matter with increased significance for the scientific community. However, a longstanding debate about the importance of variability of a system for its stability has evoked many theoretical and empirical studies. Here the authors introduce a new approach using experimental bacterial microcosms to address this question. For this study stability is defined as nonstochastic, reproducible population dynamics. The authors started with a low-diversity population and let it diversify until an adaptive event occurred. The superior genotype gradually out-competed all other competitors resulting in a selective sweep. This adaptive event served as reference “state” to test the resilience of the system. The authors investigated the reproducibility of the dynamic with competition experiments by gradual disassembly of the community. Their findings showed an increase in fitness of the superior genotype and less variation among replicate experiments with increasing complexity (number of competitors) of the system. The main implication of this study is that diversity buffers against stochastic effects.

## Introduction

As early as 1872 Darwin [1] had envisioned the critical impact that species diversity has on ecosystem dynamics. Elton [2] explicitly formulated this thought with the diversity-stability hypothesis, which proposes that ecosystem diversity is positively correlated with stability. The relationship between ecosystem functioning and species diversity is widely discussed [3–6]. While early empirical studies suggested that more diverse communities enhance ecosystem stability [2,7], subsequent ecological models indicated that diversity tends to destabilize community dynamics [8]. Since then, more realistic models have been proposed that reconcile community complexity with ecosystem stability [9,10]. Food web structure has been discussed as centrally important in the relationship between ecosystem stability and diversity [11]. If the distribution of consumer-resource interaction is skewed to weak interactions, ecosystem diversity is positively linked with stability (weak-interaction effect [12]). Nevertheless, the intrinsic complication of measuring ecosystem stability has resulted in opposing outcomes, depending on how ecosystem stability is defined [13].

The majority of experimental ecosystems have focused on assemblies of different species, often covering a range of trophic levels. Attempts to study intraspecific variation as a way to work under more controlled experimental conditions imply the main drawback: the difficulty of distinguishing intraspecific variants. Nevertheless, the importance of intraspecific variation for ecosystem functioning should not be underestimated. One particularly good example of the effect of intraspecific variation on ecological dynamics is the analysis of predator-prey cycles of a system consisting of one species in each group (rotifers and algae) [14]. The authors demonstrated that genetic diversity in the prey population (algae) significantly altered the predator-prey cycle in length and synchronisation. Other examples of the ecological impact of intraspecific variation were provided by recent studies on eelgrass diversity [15].

In this report, we focus on intraspecific variation generated in an evolving E. coli population, testing how diversity affects the evolutionary trajectory of the population. If the evolutionary trajectory is repeatable (and thus predictable), we consider the system of evolved E. coli cells to be stable. Within a recently introduced classification system of definitions for ecosystem stability [16], our use corresponds best with the term “resilience” [17]. However, rather than testing for a return to a reference state after a disturbance (the formal definition of resilience), we tested for attainment of the reference state, namely the spread of a beneficial mutation. Using a highly informative marker system, we monitored the spread of a beneficial genotype in systems with different levels of complexity.

## Results

We used experimental evolution to generate an evolved community of diverged E. coli lineages. The stability of the system was measured by the reproducibility of the dynamics of a beneficial mutation that occurred in the intact system.

**Figure 1:**
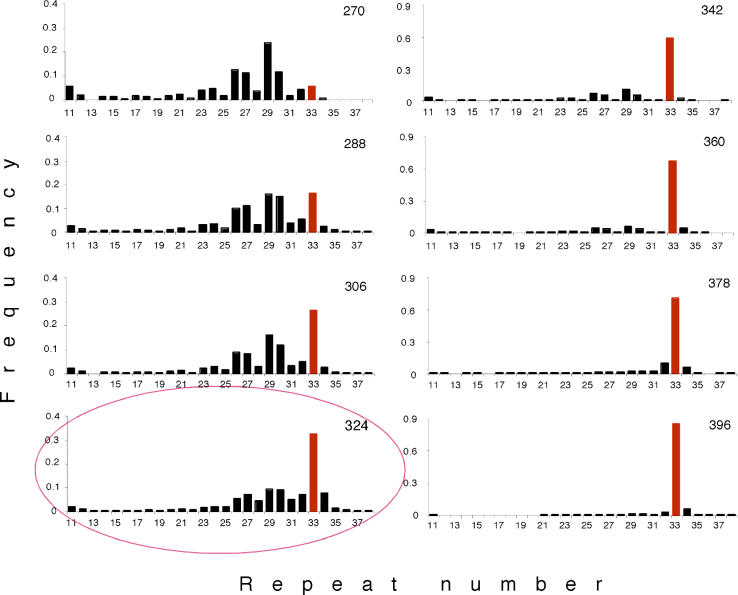
Changes in Allele Frequency at a Microsatellite Marker during the Spread of a Beneficial Mutation A “snapshot” of the allele distribution in the evolving E. coli population is shown for every eighteenth generation. The number of generations after the start of the experiment is given on the upper right corner of each graph. Bars represent the frequency of the corresponding microsatellite allele. The microsatellite allele carried by the cell with the beneficial mutation (sweeper) is shown in red. The red ellipse indicates the generation at which we isolated the cells used for the competition experiments. Note that for better resolution the scale of the y-axis has been modified between generations 324 and 342.

Beneficial mutations regularly arise in experimental E. coli populations [18,19]. We used a microsatellite marker to infer the spread of beneficial mutations in the evolving E. coli population [19]. Figure 1 shows the deterministic spread of a genotype carrying the beneficial mutation (indicated by the red bar, 33 repeats). This selective sweep was reproduced in a recent study of five independent replicate cultures [19]. Cells with (sweepers) and without (competitors) the beneficial mutation were isolated at generation 324, as at this point in time the sweeper had already reached a considerable frequency, but a large diversity of competitor cells was still present (Figure 1). Among the competitors, isolated cells differed by size of the microsatellite marker as well as by tetracycline resistance and levels of adherence, indicating that the harvested cells had already diversified (Tables S1 and S2).

For three competitor genotypes (360 experiments using the genotypes with clone numbers 902, 903, and 1139; for further details see Tables 1, S1, and S2) we performed a detailed analysis on the influence of the frequency of a given genotype on the outcome of the competition experiment. The frequency of the competitors at the onset of the competition experiments ranged from 0.04 to 0.96. Two competitors showed no frequency dependence, but for one competitor we detected a significant correlation between the starting frequency and fitness (two-tailed Spearman's Rank correlation, *r* ^2^ = 0.45, *p* ≤ 0.001, *n* = 84 [unpublished data]).

**Table 1:**
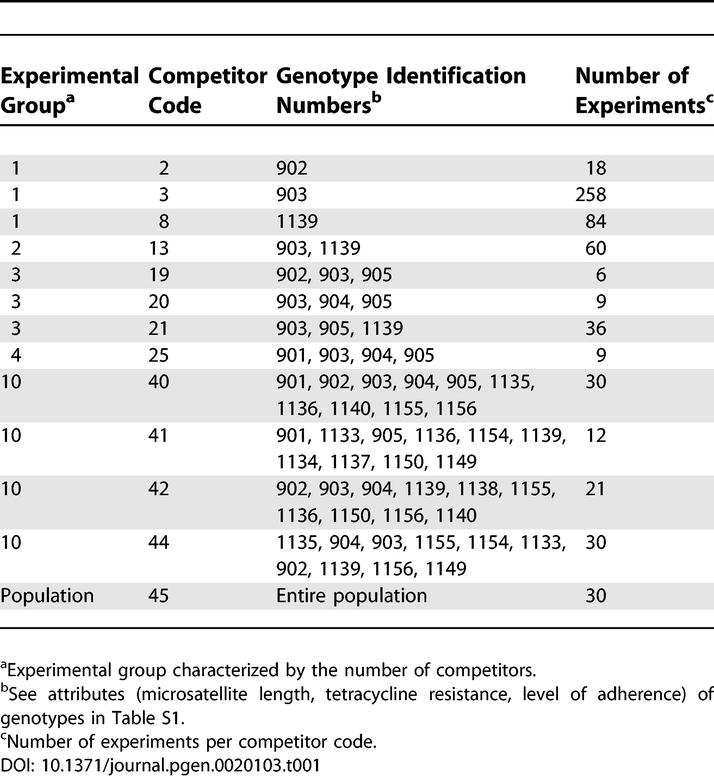
Clone Combinations of Experimental Groups

We determined the effect of system complexity by competition experiments using different levels of complexity: The extreme cases were either individual competitors (low diversity) or the whole population consisting of the entire collection of genotypes (high diversity). Intermediate levels of diversity were obtained by gradually increasing the number of competitors. Consistent with phenotypic and genotypic divergence among the competitor clones, we also found that the outcome of low diversity experiments was dependent on the genotype of the competitor cell (Tables 1 and S1). To account for this heterogeneity, we always considered the average fitness of the competitor genotypes (or combinations of genotypes; see Table 1 and Materials and Methods for more details).

**Figure 2:**
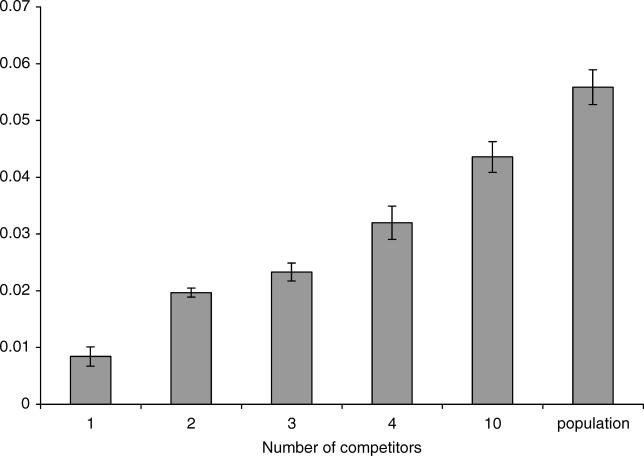
Relationship between Genetic Diversity of Competitors (Complexity) and Mean Fitness of the Clone Carrying the Beneficial Mutation (Sweeper) Fitness of the sweeper was determined by competition against a single competitor (lowest level of complexity, far left bar) increasing up to the entire population (highest level of complexity, very right bar). The number of experiments performed for each experimental group and each combination of competitors can be found in Table 1. Error bars indicate the standard deviation of the Malthusian fitness parameter determined by 100 bootstrap pseudoreplicates. The mean of the means and standard deviations of these values are plotted.

Mean fitness was significantly lower in experiments involving a single competitor than the entire population (*p* < 0.003, permutation test based on 300 replicates, Figure 2). When intermediate levels of complexity were also considered, we found a strong correlation between mean fitness and diversity (two-tailed Spearman's rank correlation, *ρ* = 0.94; *p* = 0.005; *n* = 6 [Figure 2]). Thus, the fitness of the cells carrying the beneficial mutation depends on the level of complexity of the system.

**Figure 3:**
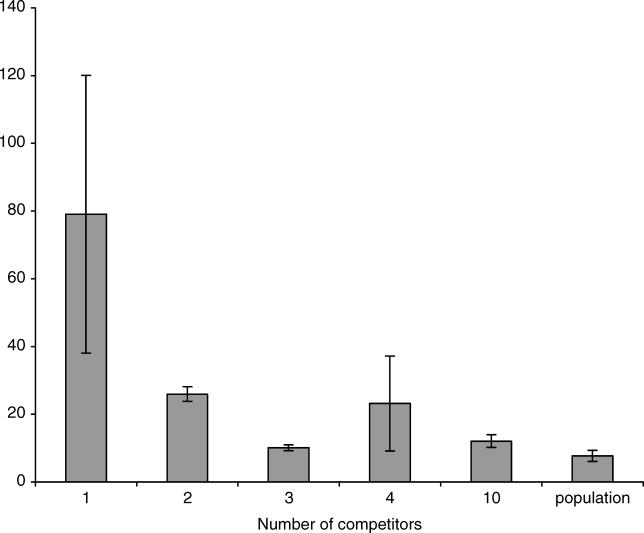
Heterogeneity among Replicate Experiments For each level of complexity (number of competitors) we determined the mean coefficient of variation of three replicate experiments. The number of experiments performed for each experimental group and each combination of competitors can be found in Table 1. Error bars indicate the standard deviation of 100 bootstrap values obtained by resampling experiments (and the corresponding coefficient of variation).

We further discovered that in 78 (~22%) of 360 of the competition experiments involving single competitors, the clone carrying the beneficial mutation was not just less fit than in higher complexity experiments but even lost (exhibited negative fitness values) against the competitor cell. To further quantify this effect, we performed three replicate experiments for each competitor and determined the heterogeneity in Malthusian fitness of the sweeper among the three replicates. We observed the lowest variation for those competition experiments with the highest complexity level (entire population) and the highest variation among replicate experiments at the lowest complexity level (one competitor genotype, *p* < 0.003, permutation test based on 300 replicates [Figure 3]). The comparison to intermediate complexity levels indicated that the decrease in heterogeneity is not linear, as the coefficient of variation for two competitors was reduced to about 30% of the single competitor experiments. Nevertheless, the highest complexity level resulted in the lowest coefficient of variation, indicating that reproducibility increases with complexity.

## Discussion

### To What Extent Are Microbial Models Suitable for Making Inferences about Ecosystem Dynamics?

Microbial models offer a variety of experimental advantages, such as short generation times, low cost, and the possibility of preserving genotypes by freezing. Several ecological issues such as succession, the diversity-stability relationship, predator-prey dynamics, the coexistence of competitors, and the coexistence of generalists and specialists are readily addressed with microbial model systems [20].

Nevertheless, it is also well understood that adaptation differs profoundly between prokaryotes and eukaryotes [21]. Although in bacteria, beneficial mutations are mainly fixed sequentially, in sexually reproducing eukaryotes, recombination allows different beneficial mutations to combine in the same genotype [22,23]. Furthermore, in general bacteria exhibit little homologous gene recombination, but do exhibit high rates of horizontal gene transfer (frequently considered an indication that a new species concept for prokaryotes is needed [24–28]), whereas sexual eukaryotes frequently reshuffle their genes but rarely acquire genes from other species [29]. Nevertheless, horizontal gene transfer should play a minor role in our single-species E. coli experiments.

Our data suggest that high diversity of (nonrecombining) genotypes favours stability. Interestingly, two recent studies on ecosystem recovery and dependence on diversity come to similar conclusions using a eukaryotic system. The authors demonstrated that a higher number of eelgrass genotypes result in significantly higher resistance against disturbance (grazing geese) [30]. Furthermore, a higher genotypic diversity of the eelgrass also resulted in an increase in the number of invertebrates after perturbation (extreme heat wave) [15]. This similarity suggests that bacterial models are probably well suited to the study of ecological processes, in particular to study the importance of intraspecific diversity.

### Diversity and Fitness

We found that mean fitness of the sweeper changed significantly in experiments involving a single competitor compared to those with more competitors. The highest level of complexity is most similar to the environment in which the beneficial mutation originated. This indicates that fitness of individual clones depends strongly on the system in which they have evolved. We think that our approach of using a coevolved community reflects real ecosystems better than randomly assembled systems, as these are thought not to be realistic [31].

If our observation is extrapolated to other systems, it may be concluded that attempts aiming to restore disturbed communities by using a small number of founder genotypes, whose individual performance is known only in systems with high complexity, may therefore not be the optimal strategy. Nevertheless, given the obvious simplification of our experimental system, further work is required to validate this conclusion.

### Does Diversity Buffer against Stochastic Effects?

The experiments involving two genotypes—the sweeper and one competitor—were found to be highly stochastic. In some experiments, the fitness of the sweeper was actually lower than that of the competitor genotype. In population genetics, such observations are attributed to stochastic effects during the early phase of a selective sweep, when the frequency of the beneficial mutation is low. If the beneficial allele reaches a higher frequency, the stochastic phase is followed by a deterministic phase at which random effects can be safely ignored. In our experiments at least 4% of the cells carried the beneficial mutation, thus a deterministic outcome of the sweep was expected (given a population size >10^6^). Further evidence against genetic drift is provided by our high-complexity experiments, which were highly reproducible despite the fact that the frequency of the sweeper was similar to that in the single-competitor experiments. Hence, we conclude that the highly stochastic outcome of the competition experiments in a low-diversity setting characterizes an intrinsic property of the experimental system: genetic diversity buffers against the stochastic outcome of the competition experiments.

What might be the basis of the buffering effect of diversity seen in our experiments? One possible explanation could be gleaned from other microcosm experiments. By reducing the number of members of the community, the balance of the system is disturbed, leading to considerable stochastic noise, possibly due to the loss of redundancy [32,33]. An alternative scenario assumes that the co-occurring competitor clones are already functionally diverged. It is conceivable that through coevolved trophic interactions the system is stabilized. In experiments with reduced complexity, such interactions are diminished, which could explain the higher stochasticity in our experiments. Previous studies on yeast and bacteria indicated that trophic interactions based on secondary metabolites can occur during the cultivation of cells derived from the same ancestor [34–36]. Interestingly, such trophic interactions either could have detrimental effects on co-occurring genotypes [37–39] or they could be utilized by community members via cross-feeding, establishing the basis for simple food webs [40–42]. While we do not know whether such trophic interactions had already emerged in our experiment, the genetic and phenotypic divergence among the competitors suggest that this possibility should be considered. Further work is required to test if the reduction of stochastic effects with an increasing number of competitor genotypes is limited to co-evolved competitors or if similar effects could be obtained by independently evolved genotypes.

Assuming that our findings from E. coli can be extrapolated to other communities, our results imply that disturbed ecosystems characterized by reduced diversity (compared to undisturbed systems, which contain more functional groups) might be more affected by stochastic effects of population dynamics than are complex (undisturbed) systems.

## Materials and Methods

### Experimental background.

Starting from a single E. coli cell we performed an evolution experiment to build a simple community consisting of diverged E. coli lineages with possible interactions at different levels. The population evolved in rich medium to avoid restrictions in adaptability due to the culture medium. Thus, the population could develop in a complex medium (environment) that fostered the possibility of a broad spectrum of niches and trophic interactions among the members of the evolving community.

### Bacterial strain, culture conditions, and detection of the adaptive event.

In brief, the evolution experiment was performed with the common laboratory strain Escherichia coli XL1 blue (*recA1 end A1 gyr A96 thi-1 hsdR17 sup E44 relA1 lac* [F′ *pro AB lacI^q^ ZΔM15 Tn10*]) (Stratagene, La Jolla, California, United States). Cells were maintained by serial transfer in 5 ml of rich medium (Lennox L Broth Base, GIBCO BRL, San Diego, California, United States) at 37 °C and 250 rpm. Every 12 h the population was diluted 1:500, allowing about nine generations per transfer. Bacterial density at transfer was ~5 × 10^8^ cells/ml. The number of generations per growth cycle (we use the variable *g* to indicate growth cycle number) was taken from [19].

E. coli cells carry a highly variable (GA)_n_ microsatellite marker. We determined the length of this marker by a restriction digest that cleaved in the sequence flanking the microsatellite. Subsequent electrophoresis separated the microsatellite alleles of different sizes [19]. The frequency of each microsatellite allele was estimated by the relative intensity of the corresponding allele.

For the competition experiments, we measured the frequency of a bacterial genotype by quantitating the intensity of the microsatellite allele associated with that genotype. The frequency change was determined by a restriction analysis at the beginning and end of the experiment. For further details on the analytical procedure, see Imhof [19].

### Isolation of clones from the sweeper lineage.

At generation 324 we isolated four clones carrying the microsatellite allele that had rapidly increased in frequency. We performed a series of competition experiments using the competitor genotypes 8, 13, and 21 (see below; Table 1), and all three clones from the sweeper lineage yielded a similar selection coefficient (Kruskal-Wallis H test, χ^2^ = 1.150, *p* = 0.765, *n* = 53; unpublished data). On the basis of these results, we concluded that no heterogeneity among the four sweeper genotypes exists; thus they were used interchangeably for the remaining experiments.

### Derivation and characterization of competitors.

A subsample of the evolving population was plated at generation 324, a few generations before the advantageous genotype was fixed, but its increase in frequency was already recognizable (Figure 1). At that stage the heterogeneity of the population was still high. Thus, sufficiently differentiated genotypes could be isolated for the consecutive competition experiments.

Based on the microsatellite allele, we categorized the isolated clones into the groups of competitors (not carrying the beneficial mutation and the microsatellite allele was different from 33 repeats) and sweepers (carrying the beneficial mutation and the 33-repeat microsatellite allele). In total, we isolated 18 competitor cells with microsatellite alleles sized between 11 and 28 repeats (Table S1).

In addition to the genetic heterogeneity at the microsatellite, we characterized tetracycline resistance and adherence (floating versus adherent cells) to test for genetic heterogeneity of the competitors (Table S2).

To account for potential heterogeneity among derivatives from the sweeper lineage, four distinct clones carrying allele 33 were isolated and characterized. We observed no phenotypic differences (for example, tetracycline resistance and levels of adherence, Table S1) among the four sweeper genotypes.

### Competition experiments.

Competing cells were grown separately for one growth cycle (about 12 h) to reach comparable physiological states. LB medium (5 ml) was inoculated with 5 μl of the sweeper and a total of 5 μl of the competitor(s), and the culture allowed to grow for one growth cycle (12 h). At that time point, we started the competition experiment by serial transfer of a 1:500 dilution every 12 h. The rest of the starting culture was harvested, and the frequency of each competitor was determined by the intensity of the microsatellite alleles (for details see [19]). At the end of the competition experiment, we determined the frequencies of the competing genotypes in the same way. Competition time was on average 45 generations. This time span was long enough to achieve unambiguous results, while the probability of new positive mutations arising and spreading to a detectable frequency was rather low (with a beneficial mutation rate *μ* = 4 × 10^−9^ per cell generation [19]). Similarly, other mutations diversifying the competitors and thus interfering with our results should have been negligible.

Competition experiments were performed between the sweeper clone carrying beneficial mutation(s) against an increasing diversity level of competitor cells. Therefore, experiments were started with single competitors and continued with different arbitrarily selected strain combinations from a total pool of 18 isolated genotypes (Table S1). As highest diversity treatment, the entire collection of diverging genotypes (represented by the entire population, consisting of more than 18 genotypes) was used (Table 1).

With 18 different competitor cells, an extremely large number of combinations is possible, preventing a systematic investigation of the effect of the genotype on the competition experiments.

First we performed a pre-test for the highest diversity treatment. In this treatment the carrier of the beneficial mutation (sweeper) competed against the entire collection of diverging genotypes (entire population) when the beneficial mutation was absent (generation 162). To ensure the absence of the beneficial mutation at generation 162, we incubated cells from this generation without adding a clone carrying the beneficial mutation. There was no increase in frequency of any of the microsatellite allele with 33 repeats (the size of the clone with the beneficial mutation) detected.

To rule out artefacts and frequency dependence effects, competition experiments were performed with the same competitor(s)/sweeper combinations on different experimental days, starting with different overnight cultures and frequencies. The number of experiments performed for each of the 13 combinations studied is given in Table 1. Each of these experiments was done in triplicate. Triplicates were started from the same culture (thus from the same overnight culture and same frequency of each competitor genotype and the sweeper) but independently cultivated, harvested, and analysed.

In none of the competition experiments was a new selective sweep detected. This observation is consistent with previous results in the same system in which adaptive events exhibited selection coefficients in the range of Malthusian fitness parameter *m* = 0.01 to 0.06. With a starting frequency of a new mutation of *P* = 1/2*N* and population size of 5 × 10^6^ at transfer it would take approximately 3,085 to 514 generations, respectively, for a new advantageous mutation to become fixed [19]. Also no substantial diversification was noted within the rather short experimental time.

Next, it can be assumed that fitness was transitive in our study, although our approach did not allow a detailed analysis of the fitness between all nonsweeping competitors. Nevertheless, a recent study with a similar experimental setting found no evidence for nontransitivity and thus validates our assumptions [43].

### Determination of fitness parameter *m.*

The Malthusian fitness parameter *m* can be determined from the frequency change of the carrier of the advantageous mutation [44]:

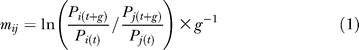
where *m*_ij_ is given per generation. *P*_i_ is the frequency of the selected lineage at time point *t* of the experiment. The frequency of genotypes that do not belong to the selected lineage *P*_j_ = 1 − *P_i_*. Competition time measured in generations is specified by *g*. The competition time differed among experiments, with a mean of 44 generations and a standard deviation of 9 (minimum = 36; maximum = 90).

### Data analysis.

As the number of experiments performed for each competitor genotype and combination of competitors differed (see Table 1) and some competitor genotypes had a strong effect on the outcome of the competition experiments (see Figure S1), we devised a special resampling strategy to account for the heterogeneity. For each level of complexity we performed multiple competition experiments for each competitor/combination of competitors. Each of these experiments was performed in triplicate (that is, it was performed in parallel, from the identical overnight culture under the identical conditions). Hence, we had a nested experimental design with multiple competition experiments each consisting of three parallel replicates.

We calculated the mean fitness *m¯* as follows:

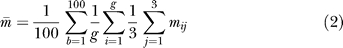



The unit of resampling is one competition experiment with the corresponding three replicate cultures (*m*_i1_ to *m*_i3_). For each of the *g* different competitors (competitor combinations) that were used for a given level of complexity, one competition experiment (with its three replicates) was selected randomly with replacement (bootstrapping [45]). We performed 100 bootstrap replications. The standard deviation was calculated accordingly. Note, that this procedure gives equal weight to each different competitor genotype (combination of genotypes).

The variance in competition experiments (reproducibility) was measured slightly differently. Rather than averaging the replicates *m*_i1_ to *m*_i3_ we calculated their coefficient of variation and averaged it over *g* different competitors (competitor combinations).

## Supporting Information

Figure S1 Mean Fitness of the Clone Carrying the Beneficial Mutation (Sweeper) against Individual Competitors and the Entire Population (46 KB PPT)

Table S1 Attributes of 22 Genotypes Isolated for the Competition Experiments (39 KB DOC)

Table S2 Criteria for Levels of Adherence (27 KB DOC)
