# Supplementary material for: E. coli Microcosms Indicate a Tight Link between Predictability of Ecosystem Dynamics and Diversity
Source: PLoS Genet. 2006 Jul 14;2(7):e103. doi: 10.1371/journal.pgen.0020103 (PMC1500808; doi:10.1371/journal.pgen.0020103)
Supplement: Supplementary file 1 [file pgen.0020103.st001.doc]

**Table S1**. Attributes of 22 genotypes isolated for the competition experiments

| **Clone #** | **No. of Repeats^a^** | **Adherence^b^** | **Tetracycline Resistance^c^** |
| --- | --- | --- | --- |
| 901 | 11 | 0 | 0 |
| 902* | 11 | 1 | 1 |
| 1137 | 11 | 0.25 | 1 |
| 1139* | 11 | 0 | 0 |
| 1149 | 11 | 0.75 | 1 |
| 903* | 14 | 0 | 0 |
| 1133 | 14 | 1 | 0 |
| 1134 | 14 | 0 | 1 |
| 905 | 20 | 1 | 1 |
| 1138 | 20 | 1 | 0 |
| 1150 | 20 | 0.25 | 0 |
| 1156 | 21 | 0.25 | 0 |
| 1136 | 24 | 1 | 0 |
| 1154 | 25 | 1 | 1 |
| 1155 | 25 | 1 | 0 |
| 904 | 27 | 0 | 0 |
| 1140 | 27 | 0.75 | 1 |
| 1135 | 28 | 0 | 1 |
| 906** | 33 | 0 | 0 |
| 907** | 33 | 0 | 0 |
| 1141** | 33 | 0 | 0 |
| 1142** | 33 | 0 | 0 |

^a^ number of the (GA) repeats of the microsatellite

^b^ level of adherence was estimated by the size of the area covered by adherent cells on the bottom of the vial versus OD_600_ of the media affected by the number of floating cells

We distinguished five categories of level of adherence, with 0 showing the lowest and 1 the highest level of adherence. *E. coli* genotypes were categorized based on two criteria, the size of the area covered by adherent cells on the bottom of the vial after 12h of cultivation and OD_600_ of the medium collected in the upper 20% of the medium in the vial (see Table S2). For each genotype, five independent classifications were made, which were 100% consistent.

^c^ assessed by plating on tetracycline plates (13 µg/ml), 0 = not resistant, 1 = resistant

* clones used for detailed single competitor studies

** clones belonging to the sweeping lineage
