# Supplementary material for: E. coli Microcosms Indicate a Tight Link between Predictability of Ecosystem Dynamics and Diversity
Source: PLoS Genet. 2006 Jul 14;2(7):e103. doi: 10.1371/journal.pgen.0020103 (PMC1500808; doi:10.1371/journal.pgen.0020103)
Supplement: Supplementary file 2 [file pgen.0020103.st002.doc]

**Table S2.** Criteria for levels of adherence

| **Adherence** | **OD_600_** | **Area covered with cells** |
| --- | --- | --- |
| 0 | >1.2 | no cells |
| 0.25 | 0.9-1.2 | few cells in the centre of the bottom |
| 0.5 | 0.6-0.9 | thin layer on entire bottom of vial |
| 0.75 | 0.3-0.6 | thick layer of cells on bottom only |
| 1 | <0.3 | thick layer of cells on bottom continuing up the side walls |
|  |  |  |
